# Fractional-Order Sensing and Control: Embedding the Nonlinear Dynamics of Robot Manipulators into the Multidimensional Scaling Method

**DOI:** 10.3390/s21227736

**Published:** 2021-11-20

**Authors:** António M. Lopes, José A. Tenreiro Machado

**Affiliations:** 1 LAETA/INEGI, Faculty of Engineering, University of Porto, Rua Dr. Roberto Frias, 4200-465 Porto, Portugal; 2 Institute of Engineering, Polytechnic of Porto, Department of Electrical Engineering, Rua Dr. António Bernardino de Almeida, 431, 4249-015 Porto, Portugal

**Keywords:** variable structure control, fractional calculus, robot manipulator, fractional sensor, multidimensional scaling, information visualization

## Abstract

This paper studies the use of multidimensional scaling (MDS) to assess the performance of fractional-order variable structure controllers (VSCs). The test bed consisted of a revolute planar robotic manipulator. The fractional derivatives required by the VSC can be obtained either by adopting numerical real-time signal processing or by using adequate sensors exhibiting fractional dynamics. Integer (fractional) VCS and fractional (integer) sliding mode combinations with different design parameters were tested. Two performance indices based in the time and frequency domains were adopted to compare the system states. The MDS generated the loci of objects corresponding to the tested cases, and the patterns were interpreted as signatures of the system behavior. Numerical experiments illustrated the feasibility and effectiveness of the approach for assessing and visualizing VSC systems.

## 1. Introduction

Variable structure systems (VSSs) [1,2] have good feasibility and robustness. As concerns system theory, variable structure controllers (VSCs) are a relevant strategy when facing systems with complex dynamics [3,4,5]. VSCs apply switching control laws to alter the system dynamics and compel the system states to slide along a cross-section named the sliding surface. The system trajectories have two distinct periods, usually called the (i) reaching and (ii) sliding mode phases. In the reaching phase, the system states are forced toward a prespecified sliding surface in finite time. Once the system states reach the sliding surface, the sliding phase initiates, and the closed-loop system states slide toward the origin along the sliding surface. During the reaching phase, the invariance of the VSC is not guaranteed and the system response is quite sensitive to perturbations. In the sliding phase, the system response remains invariant for both parametric and nonparametric uncertainties [1,6].

Mechanical manipulators exhibit strong nonlinear dynamical effects that require appropriate control algorithms. When applying VSCs with mechanical manipulators [7,8,9,10], a common practice is to approximate the dynamics of each rigid link as a first-order linear model. The imperfect implementation of high-frequency switching in VSCs results in chattering at the control responses. This effect leads to a large stress on the actuation hardware and can excite vibrations in the structure. To reduce the two effects, several schemes have been proposed, involving some kind of smoothing, namely by changing the “on-off” switching algorithm, or by complementing the VSC with some adaptive or feedforward control actions [11,12,13]. However, the first-order reference model is not well suited to the system’s intrinsic dynamics; therefore, a second-order model was proposed [2,14,15].

Fractional calculus (FC) extends the scope of the classical calculus to noninteger orders [16,17,18,19,20]. Fractional derivatives and integrals are helpful in control since they allow adapting existing integer algorithms to a fractional version with more degrees of freedom. Indeed, due its properties, FC emerged as a key tool in dynamics and control systems [21,22,23,24,25].

Embedding the FC concepts in VSCs has also been proposed [26,27,28,29,30]. Since we have the freedom of selecting the fractional order, we can take advantage of the new parameter to tune either the reference model or the control algorithm, affecting, thereby, the VSC switching action. Previous work revealed that combining FC and VSC leads to superior performance [31,32,33]. However, the algorithms require either numerical real-time signal differentiation or adequate sensors to obtain the fractional derivatives. We can cite recent works, namely Delavari et al. [28], who proposed a fractional-order sliding-mode controller to control a flexible-link manipulator, while determining the design parameters trough particle swarm optimization (PSO). Delavari and Heydarinejad [29] designed a fractional-order backstepping-sliding-mode control for a class of fractional nonlinear systems with mismatched disturbances, which were estimated using a fractional nonlinear observer. Simulation examples showed the effectiveness of the control strategy. Wang et al. [34] investigated cable-driven manipulators under lumped uncertainties and proposed an adaptive fractional control scheme based on time-delay estimation. The controller included a time-delay estimation to compensate the unknown system dynamics, a fractional nonsingular terminal sliding-mode surface to ensure high precision in the steady phase, and a reaching law with an adaptive technique to obtain fast convergence, high precision, and reduced chatter. Simulations and experiments were performed to show the effectiveness of the scheme. Zhou et al. [35] proposed a deep-convolutional-neural network-based fractional terminal sliding-mode controller for rigid manipulators. The neural network compensated the uncertainties of the system. The chattering was mitigated, and the control strategy exhibited robust performance against uncertainties and disturbances. Ma et al. [31] proposed a quaternary fractional-order sliding-mode controller with fuzzy logic system, a neural network, and an adaptive law to control the teleoperated cyberphysical system. External disturbances, modeling uncertainties, and actuator faults were considered. Xie et al. [32] addressed a coupled fractional sliding-mode control, together with an obstacle-avoidance scheme, to control a four-wheeled steerable mobile robot. A modified near-time-optimal potential function was introduced to improve collision problems. Fuzzy rules and proper adaption gains were designed to mitigate chattering. Asymptotic stability and convergence were guaranteed for the closed-loop system. Delavari and Jokar [33] presented a fractional-order active fault-tolerant controller based on an adaptive nonlinear observer to detect, estimate, and compensate faults of a knee joint orthosis. The controller was based on fractional-order sliding-mode control, while the switching term was designed using fractional-order interval type-2 fuzzy logic. The strategy was proven to reduce modeling issues and chattering. Other examples can be found in [36,37,38].

Several researchers proposed a variety of fractional controllers both in the discrete-time [39,40,41] and frequency [42,43,44] domains. The implementation of such controllers requires the use of approximation algorithms to obtain the fractional derivatives or the implementation of sensors exhibiting fractional dynamics. The construction of these sensors can take advantage of the advanced fabrication techniques of microelectromechanical systems (MEMSs) [45], allowing, therefore, directly obtaining the fractional derivatives [46].

We have well-known indices to assess the performance of controlled systems. However, in general, a single criterion is not sufficient to capture the dynamic details. Therefore, characterizing the system behavior is a multidimensional problem that can be tackled with recent computational tools [47,48]. Dimensionality reduction [49] plays a key role, since the data often exhibit a multidimensional nature. Dimensionality-reduction-based schemes try to preserve in lower-dimensional representations the information present in the original datasets. They include linear methods, such as classic multidimensional scaling (MDS) [50], principal component [51], canonical correlation [52], linear discriminant [53], and factor analysis [54], as well as nonlinear approaches, such as nonclassic MDS or Sammon’s projection [55], isomaps [56], Laplacian eigenmaps [57], diffusion maps [58], t-distributed stochastic neighbor embedding [59], and uniform manifold approximation and projection [60]. Besides dimensionality reduction, these algorithms often allow direct information visualization.

Hereafter, we considered the MDS technique to evaluate and visualize the performance of fractional-order VSC. The test bed adopted consisted of a revolute planar robotic manipulator. The fractional derivatives required by the controller can be implemented either by numerical methods or by using sensors exhibiting fractional dynamics. Integer (fractional) VCS and fractional (integer) sliding mode combinations, with different design parameters, were studied. The system states for a number of test cases were obtained and compared by means of two alternative distance metrics, namely in time or in frequency. The information was input into the MDS. The algorithm then generated the loci of objects where each point corresponded to one test case. The objects patterns were interpreted as signatures of the system behavior. Several numerical experiments illustrated the effectiveness of the approach.

The paper has four sections. Section 2 addresses the mathematical background and key concepts useful in the remainder of the paper. Section 3 analyzes the dynamics of the VSC system by means of MDS, while adopting the integer VSC and fractional sliding mode (IVSC-FSM) and the fractional VSC and integer sliding mode (FVSC-ISM). The analysis is performed either in the time or frequency domain. Finally, Section 4 presents the main conclusions.

## 2. Preliminaries

### 2.1. Fractional Integrodifferential Operators

The Grünwald–Letnikov (GL) fractional operator of order α∈R on y(t), denoted as ${}_{a}^{GL}D_{t}^{\alpha }y\left(t\right)$, is given by [61]:

(1) $$\begin{array}{c} {}_{a}^{GL}D_{t}^{\alpha }y\left(t\right)=\lim_{h\to 0}h^{-\alpha }\sum_{k=0}^{\left[\frac{t-a}{h}\right]}{\left(-1\right)}^{k}\left(\begin{array}{c} \alpha \\ k \end{array}\right)y\left(t-kh\right),\;\alpha >0, \end{array}$$

where [·] denotes the integer part, *h* is the time increment, and {t,a}∈R (t>a) are the upper and lower limits of the “differintegral” operation, respectively.

In signal processing and control, the GL definition (1) can be approximated numerically using [62,63]:

(2) $$\begin{array}{cc} {}_{a}^{GL}D_{t}^{\alpha }y\left(t\right) & \approx {\;}_{\left(t-L\right)}^{GL}D_{t}^{\alpha }y\left(t\right)=T^{-\alpha }\sum_{k=0}^{M(t)}{\left(-1\right)}^{k}\left(\begin{array}{c} \alpha \\ k \end{array}\right)y\left(t-kT\right)=T^{-\alpha }\sum_{k=0}^{M(t)}c_{k}^{\left(\alpha \right)}y\left(t-kT\right), \end{array}$$

where *T* is the sampling period, *L* corresponds to the “memory length”, and M(t)=min{[t/h],[L/h]}.

The binomials $c_{k}^{\left(\alpha \right)}$ are computed by [62]:

(3) $$c_{k}^{\left(\alpha \right)}=\left(1-\frac{1+\alpha }{k}\right)c_{k-1}^{\left(\alpha \right)},\;c_{0}^{\left(\alpha \right)}=1.$$

The “memory length” *L* is often chosen considering:

(4) $$L\geq \frac{1}{\delta_{0}^{2}\Gamma \left(\alpha \right)},$$

where Γ is the gamma function and $\delta_{0}$ represents the maximum allowable error:

(5) $$\delta_{0}=\frac{{|}_{a}^{GL}D_{t}^{\alpha }y\left(t\right)-{\;}_{\left(t-L\right)}^{GL}D_{t}^{\alpha }y\left(t\right)|}{P},\;P=\max_{\left[0,\infty \right]}\left|y\left(t\right)\right|.$$

In many practical applications, we consider a=0 and adopt $D_{t}^{\alpha }$ to denote the generalized “differintegral” operator.

Alternatively, we can use the approximation:

(6) $$\begin{array}{c} Z\left\{D^{\alpha }y\left(t\right)\right\}\approx T^{-\alpha }\sum_{k=0}^{M(t)}{\left(-1\right)}^{k}\left(\begin{array}{c} \alpha \\ k \end{array}\right)y\left(t-kT\right)z^{-k}Z\left\{y\left(t\right)\right\}={\left(\frac{1-z^{-1}}{T}\right)}^{\alpha }Z\left\{y\left(t\right)\right\},\;\;z\in C, \end{array}$$

where Z{·} denotes the Z transform.

Equation (6) gives the *s* → *z* Euler approximation, but we find often other algorithms in controller design, such as the so-called Tustin or trapezoidal scheme. The Euler and Tustin conversion techniques can be generalized in the scope of FC to:

(7) $$s^{\alpha }\approx {\left[\frac{1}{T}\left(1-z^{-1}\right)\right]}^{\alpha }={\left[\Psi_{0}\left(z^{-1}\right)\right]}^{\alpha },$$

(8) $$s^{\alpha }\approx {\left(\frac{2}{T}\frac{1-z^{-1}}{1+z^{-1}}\right)}^{\alpha }={\left[\Psi_{1}\left(z^{-1}\right)\right]}^{\alpha },$$

where ${\left[\Psi_{0}\left(z^{-1}\right)\right]}^{\alpha }$ and ${\left[\Psi_{1}\left(z^{-1}\right)\right]}^{\alpha }$ are approximation functions of zero and one order, respectively.

To obtain rational expressions, we need to truncate the Taylor series or Padé fraction.

The two approximations ${\left[\Psi_{0}\left(z^{-1}\right)\right]}^{\alpha }$ and ${\left[\Psi_{1}\left(z^{-1}\right)\right]}^{\alpha }$ can be averaged with weights *p* and 1−p, respectively, resulting in:

(9) $$s^{\alpha }\approx \Psi_{av}\left[z^{-1};\left(p,\alpha \right)\right]=p{\left[\Psi_{0}\left(z^{-1}\right)\right]}^{\alpha }+\left(1-p\right){\left[\Psi_{1}\left(z^{-1}\right)\right]}^{\alpha }.$$

For instance, the case $p=\frac{3}{4}$ corresponds to the Al-Alaoui operator [64,65].

### 2.2. Variable Structure Control

When adopting the VSC in a manipulator, the *k*-th link (k=1,⋯,K) is induced to mimic a first-order reference:

(10) $$\sigma_{k}={\dot{e}}_{k}+\lambda_{k}e_{k}=0,$$

(11) $$e_{k}=\theta_{dk}-\theta_{k},$$

with $\left\{\theta_{dk},{\dot{\theta }}_{dk}\right\}$ and $\left\{\theta_{k},{\dot{\theta }}_{k}\right\}$ denoting the desired and the true position and velocity of the *k*-th joint, respectively, $\sigma_{k}$ standing for the switching variable, and $e_{k}$ representing the position error. The expression $s+\lambda_{k}=0$ has an eigenvalue, $\lambda_{k}\in R$, that characterizes the sliding phase dynamics.

The VSC produces a control action $T_{k}\left(t\right)$ that forces the robot to mimic the reference (10). Often, the VSC follows an algorithm of the type:

(12) $$T_{k}\left(t\right)=T_{k}\left[\mathrm{sgn}\left(\sigma_{k}\right)\right],$$

where the function $\mathrm{sgn}\left(\cdot \right)$ returns the sign of its argument. When the VSC satisfies the condition:

(13) $$\sigma_{k}\dot{\sigma_{k}}<0,$$

convergence is guaranteed.

In [66], it was observed that such a strategy leads to stringent requirements because the first-order model (10) allows discontinuous trajectories in the phase plane. However, mechanical manipulators have inertias that lead to the continuous evolution of the links’ positions and velocities. Therefore, a first-order model requires high joint torques during transients. In practice, torques cannot be infinite, and thus, the phase plane trajectories are continuous. However, the demanding torque requirements saturate the robot actuators, resulting in a longer reaching phase, which is sensitive to perturbations. Therefore, a second-order model was proposed to mitigate these problems [14]:

(14) $$\sigma_{k}=\ddot{e_{k}}+2\zeta_{k}\omega_{nk}{\dot{e}}_{k}+\omega_{nk}e_{k}=0,$$

where $\zeta_{k}$ denotes the coefficient of damping and $\omega_{nk}$ stands for the undamped natural frequency. If the corresponding Laplace equation $s^{2}+2\zeta_{k}\omega_{nk}s+\omega_{nk}^{2}=\left(s+\lambda_{k1}\right)\left(s+\lambda_{k2}\right)=0$ has negative real roots $\lambda_{k1}$ and $\lambda_{k2}$, then (14) yields an overdamped or a critically damped behavior.

The model (10) leads to a single trajectory, while (14) always gives a continuous trajectory passing through any initial condition. Therefore, the reaching period is avoided and chattering is attenuated. Indeed, when perturbations arise, the actual robot trajectory moves away from the desired one. If a first-order sliding model is used, then the controller reacts, providing opposite phase plane trajectories towards the desired one. As some delay is inherent to the digital control, a “switching” between the curves arises, originating chattering. Using second-order curves, there is always a trajectory containing a given state, and after a perturbation, the system is not forced to follow the initial trajectory. Instead, it will follow a new one that contains the present state. As a result, the controller uses a new curve, almost parallel with the previous one, passing through the actual phase plane point. To accomplish this, the algorithm requires a second-order derivative, that is to say, it requires acceleration sensors (for more details, see [14]).

To avoid this problem, introducing an integral action was also proposed, giving rise to the reference [67]:

(15) $$\sigma_{k}={\dot{e}}_{k}+2\zeta_{k}\omega_{nk}e_{k}+\omega_{nk}^{2}\int_{0}^{t}e_{k}d\tau =0,$$

and the Laplace expression $\frac{s^{2}+2\zeta_{k}\omega_{nk}s+\omega_{nk}^{2}}{s}$, with two zeros and one pole at s=0.

In [68], the problem was rewritten in the scope of FC with the reference model (10) formulated as:

(16) $$\sigma_{k}=D^{\alpha +1}e_{k}+\lambda_{k}^{\alpha }D^{1}e_{k}+\lambda_{k}D^{\alpha }e_{k}+\lambda_{k}^{\alpha +1}e_{k}=0.$$

The characteristic polynomial is now $\left(s+\lambda_{k}\right)\left(s^{\alpha }+\lambda_{k}^{\alpha }\right)$, and the fractional order of −1≤α≤1 was explored.

**Figure 1:**
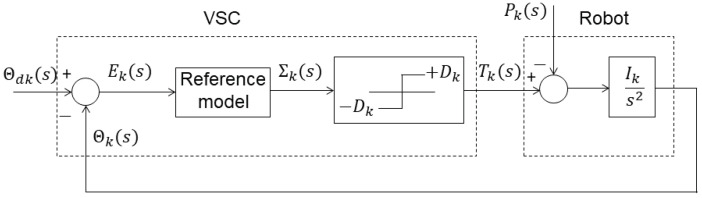
Block diagram of a VSC-controlled robot link.

**Figure 2:**
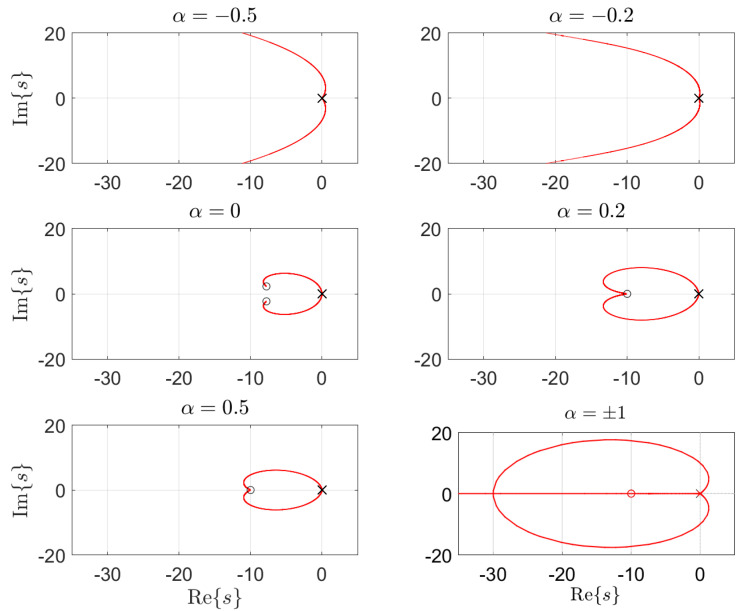
The root locus for various values of α.

Equation (16) is an “interpolation” between (14) and (15). We verified that: (i) for each robot link, Expression (16) has two zeros or two zeros and one pole, for α>0 or α<0, respectively; (ii) the dynamics of the manipulator, from torque to position, presents two poles; (iii) coupling phenomena can be regarded as perturbations, $p_{k}$ (see Figure 1). The location of the robot poles varies considerably [69], and the VSC adapts the gain to guaranty that the system’s global dynamics follows the desired reference. Figure 2 sketches the root locus of the unit-feedback VSC-controlled robot links for various values of α [70,71]. The zeros and poles of the reference model must comply with the nature of the system dynamics. Expression (16) along with the manipulator dynamics establishes a compromise between zeros and poles, yielding a perceptive reference model. However, we verified that (16) requires either numerical fractional-order differentiation or fractional-order sensors.

### 2.3. Fractional-Order Sensor

In the classical form, an accelerometer measures the second time derivative of the displacement of a rigid body. The sensor includes a moving mass that is interconnected with a casing by one spring and one damper. Therefore, the motion of the mass with respect to the casing is able to capture the acceleration of the body.

Modern accelerometers adopt MEMSs. These sensors synthesize mechanical and electrical components in a small-scale semiconductor. MEMS technology allows integrating the three mechanical elements of the device with displacement sensors and electronics.

A uni-axial accelerometer is modeled as:

(17) $$M\ddot{y}+B\dot{y}+Ky=M\ddot{u},$$

where *M*, *B*, and *K* represent the mass, damper, and spring. The motion of *M* with respect to the casing is denoted by *y* and the absolute displacement of the casing by *u*.

Applying the Laplace transform, we obtain the transfer function:

(18) $$L\left\{g\left(t\right)\right\}=G\left(s\right)=\frac{U(s)}{Y(s)}=\frac{Ms^{2}}{Ms^{2}+Bs+K}.$$

Equation (17) can be generalized to a fractional order as:

(19) $$MD^{\gamma }y+BD^{\lambda }y+KD^{\nu }y=MD^{\gamma }u,\;\gamma >\lambda >\nu ,$$

yielding the transfer function:

(20) $$G\left(s\right)=\frac{U(s)}{Y(s)}=\frac{Ms^{\gamma }}{Ms^{\gamma }+Bs^{\lambda }+Ks^{\nu }}.$$

For γ=2, λ=1, and ν=0, Equations (19) and (20) yield the dynamics of the classical accelerometer. However, (19) and (20) have no known physical meaning, and their implementation requires fractional elements that are presently not available or are unfeasible [72]. Indeed, (19) and (20) imply the fractionalization of Newton’s second law, and its meaning is somewhat controversial. On the one hand, it is straightforward to generalize (17) and (18) from integer to fractional if we adopt an abstract perspective. However, on the other hand, we do not have a guarantee of the feasibility and, furthermore, that the physical properties remain of the same type. For instance, while for the standard mass, we have $F\left(s\right)=Ms^{2}U\left(s\right)$, with F(s) denoting force, meaning it has the property of being undeformable, there is no guarantee that the fractional mass, with model $F\left(s\right)=Ms^{\gamma }U\left(s\right)$, has the same behavior.

In [45], a modular *N*-stage cascade fractional sensor was proposed. Each *k*-th stage, k=1,⋯,N, comprises the standard elements {mass, spring, damper}, represented by $\left\{M_{k},K_{k},B_{k}\right\}$. The displacement of $M_{k}$ relative to the previous stage k−1 is denoted by $y_{k}$, while the external casing of the sensor corresponds to k=0. The variables *u* and $z_{k}=u-\sum_{p=1}^{k}y_{p}$ stand for the displacements of the device casing and $M_{k}$, respectively, with respect to the external inertial frame.

The system model, having *u* as the input and $y_{1}$ as the output, is expressed in the Fourier domain as:

(21) $$G_{N}\left(j\omega \right)=\frac{Y_{1}\left(j\omega \right)}{U(j\omega )}=Z^{-1}\left(j\omega \right)\cdot Z_{1}\left(j\omega \right),$$

where ω=2πf is the angular frequency (*f* is the frequency) and Z(jω) is given by:

(22) $$\begin{array}{cc} Z & \left(j\omega \right)=Z_{1}\left(j\omega \right)+\\ & \frac{1}{Y_{1}\left(j\omega \right)+\frac{1}{Z_{2}\left(j\omega \right)+\frac{1}{\;\;\;\;\;\;\;\frac{\ldots }{Y_{N-1}\left(j\omega \right)+\frac{1}{Z_{N}\left(j\omega \right)+\frac{1}{Y_{N}\left(j\omega \right)}}}}}}, \end{array}$$

with $Z_{k}\left(j\omega \right)=\frac{j\omega }{j\omega B_{k}+K_{k}}$ and $Y_{k}\left(j\omega \right)=j\omega M_{k}$.

For the case of selecting the parameters recursively, $M_{k+1}=\eta M_{k}$, $B_{k+1}=\epsilon B_{k}$ and $K_{k+1}=\kappa K_{k}$, $\eta ,\epsilon ,\kappa \in R^{+}$, we obtain [43,73,74,75]:

(23) $$|G_{N}\left(j\omega \right)|\propto \omega^{\alpha },\;\arg\left\{G_{N}\left(j\omega \right)\right\}=\frac{\alpha \pi }{2},$$

(24) $$\alpha \approx \frac{\log(\epsilon )}{\log(\eta )+\log(\epsilon )}.$$

The interval for which $G_{N}\left(j\omega \right)$ exhibits fractional dynamics is established by the relationships $|j\omega |<\frac{B_{1}}{M_{1}}$ and $\left|j\omega \right|\ll \frac{K_{1}}{B_{1}}$. We note that for η>1, ϵ>1, and $\frac{\eta }{\kappa }>1$, the influence of $K_{k}$ for the fractional order α is minimal [43,74,75]. Nonetheless, the recursive dependence revealed problems for small α, with $\arg\left\{G_{N}\left(j\omega \right)\right\}$ oscillating. This problem limits the fractional behavior to the range 0≤α≤1, and an alternative computational procedure was proposed. Indeed, the determination of the values of the elements $\left\{M_{k},B_{k},K_{k}\right\}$, k=1,⋯,N, can be viewed as an optimization problem [45] with objective function:

(25) $$I=\left|b\cdot \frac{\pi }{2}-\langle \arg\left\{G_{N}\left(j\omega \right)\right\}\rangle \right|,\;\omega \in \left[\omega_{1},\omega_{2}\right],$$

with *b* representing the power-law $\left|G_{N}\left(j\omega \right)\right|\propto \omega^{b}$, where b∈R, and $\langle \arg\left\{G_{N}\left(j\omega \right)\right\}\rangle$ denotes the mean of $\arg\left\{G_{N}\left(j\omega \right)\right\}$, $\omega \in [\omega_{1},\omega_{2}]$. The extreme frequency values of the zeros and poles of $G_{N}\left(j\omega \right)$ impose the frequency bandwidth $\omega \in [\omega_{1},\omega_{2}]$.

It should be noted that in this algorithm, we did not choose the fractional order, nor $\left\{\omega_{1},\omega_{2}\right\}$ in advance. The numerical scheme involves running the optimization procedure a number of times and characterizing each solution through the indices {I,σ,Ω}, where *I* is the fitness function value, σ stands for the standard deviation of $\arg\{G_{N}\left(j\omega \right)\}$ in the interval ω, and $\Omega =\log\frac{\omega_{1}}{\omega_{2}}$. The user has to choose the solution taking into account the indices.

**Figure 3:**
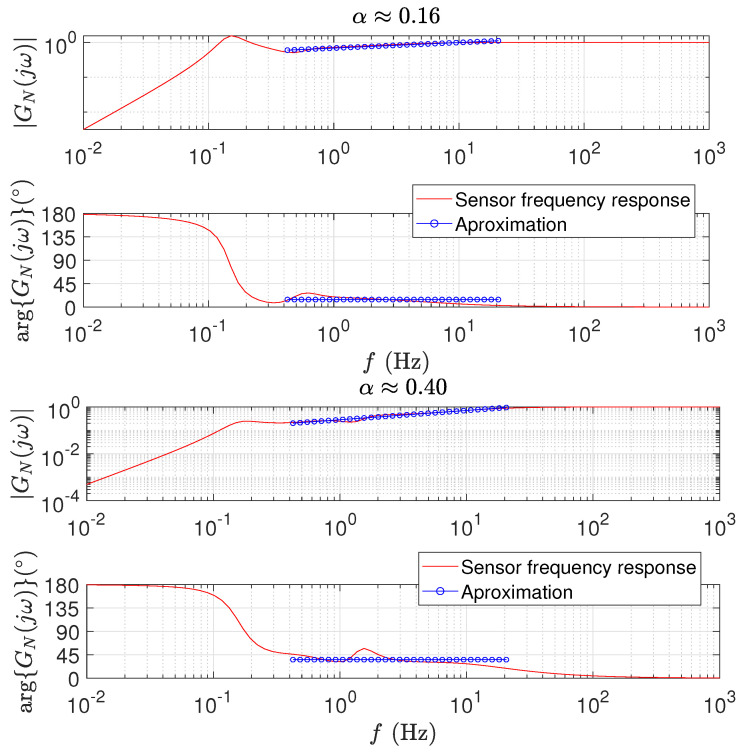
The Bode diagrams of a 6-stage fractional sensor, yielding the fractional orders: (**top**) α=0.16; (**bottom**) α=0.40.

Figure 3 illustrates the frequency response of a six-stage fractional sensor. The results correspond to two instances synthesized by a PSO algorithm with a population of twenty elements and a number of iterations equal to five, yielding the fractional orders α={0.16,0.40}. The initialization was random in the interval $\left\{M_{k},B_{k},K_{k}\right\}\in \left[0,1\right]$. The values of the sensor parameters are listed in Table 1.

**Figure 4:**
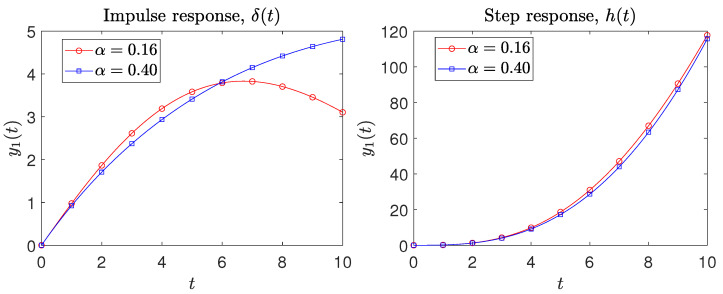
The sensor time responses $y_{1}(t$) to the input signals u(t)={δ(t),h(t)}.

Figure 4 depicts the sensor time responses $y_{1}(t$) to the Dirac and Heaviside inputs u(t)={δ(t),h(t)}. We verify, (i) in Figure 3, a good fit to the ideal responses, within the interval $\omega =[\omega_{1},\omega_{2}]$, and (ii) in Figure 4, a good behavior for the initial transient, in particular for the Heaviside input.

### 2.4. The Multidimensional Scaling Technique

MDS is a numerical procedure adopted to reduce the dimensionality and visualize high-dimensional datasets.

Let $v_{i}$, i=1,⋯,N, be objects in a space with *L* dimensions. First, we choose a distance $d(v_{i},v_{j})$, i,j=1,⋯,N, between the pairs of objects *i* and *j*, and calculate a dissimilarity matrix $D=[d\left(v_{i},v_{j}\right)]$. Then, we feed the MDS with D. The algorithm finds the coordinates of the objects, $z_{i}$, in an embedding *P*-dimensional space (P≤L) that minimize a fitness function. The result is a matrix $Z=[\hat{d}\left(z_{i},z_{j}\right)]$ that approximates D. Often, the stress cost function, S, is used as the fitness function:

(26) $$S={\left[\sum_{i<j}{\left[d\left(v_{i},v_{j}\right)-\hat{d}\left(z_{i},z_{j}\right)\right]}^{2}\right]}^{\frac{1}{2}}.$$

Nevertheless, other criteria are possible, such as the Sammon function:

(27) $$S={\left[\frac{\sum_{i<j}{\left[d\left(v_{i},v_{j}\right)-\hat{d}\left(z_{i},z_{j}\right)\right]}^{2}}{\sum_{i<j}{\left[d(v_{i},v_{j})\right]}^{2}}\right]}^{\frac{1}{2}}.$$

The MDS results were evaluated by comparing the object representations in the original and the embedding spaces. This involved the generation of the Shepard diagram, which relates $d(v_{i},v_{j})$ versus $\hat{d}\left(z_{i},z_{j}\right)$, and the stress map, which depicts S versus *P*.

A number of distances $d(v_{i},v_{j})$ are possible to construct D [76]. Here, we adopted $\left\{d_{1},d_{2}\right\}$ to quantify the dissimilarities in pairs (i,j) of objects that possess real and imaginary components. As such, the *i*-th object is represented by a matrix of dimension L×2, vi=[Re{vi1},⋯,Re{viL}T,[Im{vi1},⋯,Im{viL}]T], where Re {·} and Im {·} represent the real and imaginary parts. The distances $\left\{d_{1},d_{2}\right\}$ are given by:

(28) d1(vi,vi)=arccos∑l=1LRe{vil}Re{vjl}+∑l=1LIm{vil}Im{vjl}∑l=1LRe{vil}2+Im{vil}2∑l=1LRe{vjl}2+Im{vjl}2,

(29) d2(vi,vj)=∑l=1L[Re{vil}−Re{vjl}]2∑l=1LRe{vil}2+∑l=1LRe{vjl}2−∑l=1LRe{vil}Re{vjl}+∑l=1L[Im{vil}−Im{vjl}]2∑l=1LIm{vil}2+∑l=1LIm{vjl}2−∑l=1LIm{vil}Im{vjl}.

It should be noted that using (28) and (29), the objects (i,j) can be compared in the time or in the frequency domain. In the time domain, the vectors $v_{i}$ and $v_{j}$ have real components, and the distances $\left\{d_{1},d_{2}\right\}$ yield the standard {arccosine, Jaccard} [76]. In the frequency domain, the vectors $v_{i}$ and $v_{j}$ have complex components, and Expressions (28) and (29) are calculated directly.

## 3. Multidimensional Analysis and Visualization of Variable Structure Control

The dynamics of a planar *K*-link manipulator can be expressed as:

(30) $$J\left(\theta \right)\ddot{\theta }+C\left(\theta ,\dot{\theta }\right)+G\left(\theta \right)=T,$$

where J(θ) represents the K×K matrix of inertial terms, $C(\theta ,\dot{\theta })$ and G(θ) stand for the K×1 vectors of Coriolis and centripetal force and gravitational components, respectively, and T corresponds to the K×1 vector of torques that act on the manipulator links.

We adopted as test bed a manipulator comprising two revolute joints (K=2) and the dynamics described in [7,8]. Therefore, we have:

(31) $$J\left(\theta \right)=\left[\begin{array}{cc} 15.75+10\;\cos\left(\theta_{2}\right) & 4+5\;\cos\left(\theta_{2}\right)\\ 4+5\;\cos\left(\theta_{2}\right) & 9 \end{array}\right],$$

(32) $$C\left(\theta ,\dot{\theta }\right)=\left[\begin{array}{c} -\left(5{\dot{\theta }}_{2}+10{\dot{\theta }}_{1}\right)\sin\left(\theta_{2}\right){\dot{\theta }}_{2}\\ 5\;\sin\left(\theta_{2}\right){\dot{\theta }}_{1}^{2} \end{array}\right],$$

(33) $$G\left(\theta \right)=\left[\begin{array}{c} 66.15\;\cos\left(\theta_{1}\right)+49\;\cos\left(\theta_{1}+\theta_{2}\right)\\ 49\;\cos\left(\theta_{1}+\theta_{2}\right) \end{array}\right].$$

In the experiments, we considered the manipulator moving from an initial state given by [7,8]:

(34) $${\left[\theta_{1}\left(0\right),\theta_{2}\left(0\right)\right]}^{T}={\left[-2.784,-1.204\right]}^{T},$$

(35) $${\left[{\dot{\theta }}_{1}\left(0\right),{\dot{\theta }}_{2}\left(0\right)\right]}^{T}={\left[0,0\right]}^{T},$$

to a final one described by:

(36) $${\left[\theta_{1}\left(\infty \right),\theta_{2}\left(\infty \right)\right]}^{T}={\left[0,0\right]}^{T},$$

(37) $${\left[{\dot{\theta }}_{1}\left(\infty \right),{\dot{\theta }}_{2}\left(\infty \right)\right]}^{T}={\left[0,0\right]}^{T}.$$

To approximate the fractional derivative, we used the Al-Alaoui operator and $T=10^{-4}$ s. In the reference model, we considered $\lambda_{k}=10$ (k=1,2), and in the control action, we adopted the saturation torque values $D_{1}=200,D_{2}=100$.

### 3.1. Integer Variable Structure Control and Fractional Sliding Mode

We start by adopting the IVSC-FSM. We considered that the VSC generates a control action given by a proportional function with a threshold saturation value:

(38) $$T_{k}^{VSS}=\left\{\begin{array}{cc} +D_{k}, & \sigma_{i}>\delta_{k}\\ \frac{\sigma_{k}}{\delta_{k}}D_{k}, & -\delta_{k}\leq \sigma_{k}\leq \delta_{k}\\ -D_{k}, & \sigma_{k}<-\delta_{k} \end{array}\right.,$$

(39) $$T_{k}=T_{k}^{VSS}.$$

The set of tests consists of adopting varying fractional orders $\alpha =\{\alpha_{1},\cdots ,\alpha_{q},\cdots \alpha_{Q}\}$, $\alpha_{q}\in \left[-0.5,1\right]$, and the amplitude of the proportional band $\delta_{k}=\delta$ (k=1,2), with $\delta =\{\delta_{1},\cdots ,\delta_{q},\cdots \delta_{Q}\}$, and $\delta_{q}\in \left[10^{-4},10^{1}\right]$. For both parameters, Q=20 equidistant values were considered, yielding N=20×20 test cases in total.

For each test case, we gathered the sampled state $\theta \left(t_{r}\right)=\left[\theta_{1}\left(t_{r}\right),\theta_{2}\left(t_{r}\right)\right]$ and $\dot{\theta }\left(t_{r}\right)=\left[\dot{\theta_{1}}\left(t_{r}\right),\dot{\theta_{2}}\left(t_{r}\right)\right]$, $t_{r}\in \left[0,20\right]$ s, which yields state vectors of 20,001 dimensions. The data were then organized in an N×L=(20×20)×(4×20,001)-dimensional array:

(40) $${\tilde{W}}^{\left(t\right)}=\left[\begin{array}{cccc} \theta_{11}^{T}\left(t_{r}\right) & \theta_{21}^{T}\left(t_{r}\right) & {\dot{\theta }}_{11}^{T}\left(t_{r}\right) & {\dot{\theta }}_{21}^{T}\left(t_{r}\right)\\ \cdots & \cdots & \cdots & \cdots \\ \theta_{1i}^{T}\left(t_{r}\right) & \theta_{2i}^{T}\left(t_{r}\right) & {\dot{\theta }}_{1i}^{T}\left(t_{r}\right) & {\dot{\theta }}_{2i}^{T}\left(t_{r}\right)\\ \cdots & \cdots & \cdots & \cdots \\ \theta_{1N}^{T}\left(t_{r}\right) & \theta_{2N}^{T}\left(t_{r}\right) & {\dot{\theta }}_{1N}^{T}\left(t_{r}\right) & {\dot{\theta }}_{2N}^{T}\left(t_{r}\right) \end{array}\right].$$

We normalized the array ${\tilde{W}}^{\left(t\right)}$ by the average and standard deviation, μ(·) and σ(·), respectively, so that numerical saturation was avoided. Therefore, the columns of the matrix ${\tilde{W}}^{\left(t\right)}$, denoted by ${\tilde{u}}_{l}^{\left(t\right)}$, with l=1,⋯,L, are recalculated as:

(41) $$u_{l}^{\left(t\right)}=\frac{{\tilde{u}}_{l}^{\left(t\right)}-\mu \left({\tilde{u}}_{l}^{\left(t\right)}\right)}{\sigma ({\tilde{u}}_{l}^{\left(t\right)})},$$

and a normalized array $W^{\left(t\right)}$ is obtained. Afterwards, the rows of $W^{\left(t\right)}$, denoted by $v_{i}^{\left(t\right)}$, with i=1,⋯,N, are used to compute the dissimilarity matrices $D_{h}=\left[d_{h}\left(v_{i}^{\left(t\right)},v_{j}^{\left(t\right)}\right)\right]$, i,j=1,⋯,400, with h=1,2, that feed the MDS.

**Figure 5:**
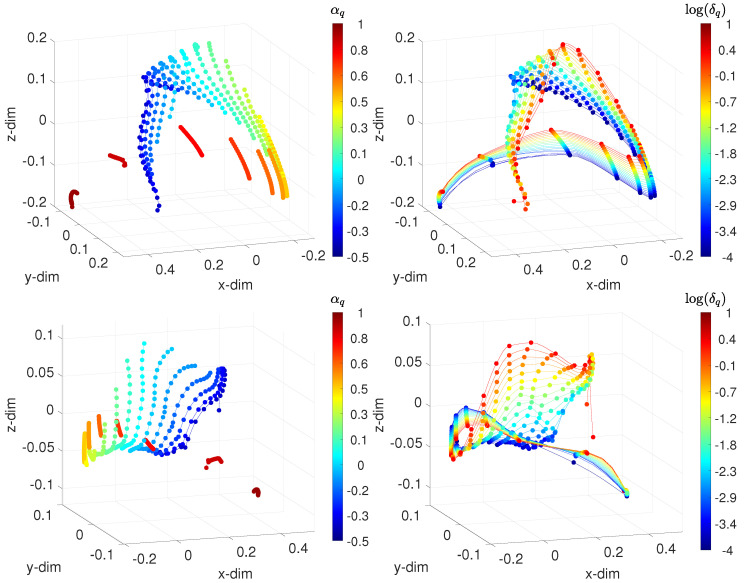
The 3-dim MDS locus for the IVSC-FSM, assessing the time-domain behavior, with the distances: (**top**) Arccosine $d_{1}\left(v_{i}^{\left(t\right)},v_{j}^{\left(t\right)}\right)$; and (**bottom**) Jaccard $d_{2}\left(v_{i}^{\left(t\right)},v_{j}^{\left(t\right)}\right)$. The points represent the test cases. On the left, lines connect points of constant δ, and each color corresponds to points of constant α. On the right, lines connect points of constant α, and each color corresponds to points of constant δ. The fractional order $\alpha_{q}\in \left[-0.5,1\right]$, and the width of the band $\delta_{q}\in \left[10^{-4},10^{1}\right]$.

Figure 5 sketches the 3-dim loci generated when adopting the arccosine distances and Jaccard distances $d_{1}\left(v_{i}^{\left(t\right)},v_{j}^{\left(t\right)}\right)$ and $d_{2}\left(v_{i}^{\left(t\right)},v_{j}^{\left(t\right)}\right)$. The points, which represent the test cases, are linked by means of lines of constant δ (or α) values, while each color corresponds to one value of α (or δ). When the parameters varied, we obtained patterns that characterized the dynamics of the controlled system. For constant and low α, as δ grows, the loci describe a path that reaches an inflection zone and change direction. The round trips are identical for low values of α. As α increases, the inflection tends to vanish, and the locus becomes more insensitive to δ. For δ constant, we also verified the emergence of patterns, being similar to each other for all values of α.

The behavior of a system can also be assessed in the frequency domain. Therefore, we computed the Fourier transform of the state vectors $F\left\{\theta \left(t_{r}\right)\right\}=\left[F\left\{\theta_{1}\left(t_{r}\right)\right\},F\left\{\theta_{2}\left(t_{r}\right)\right\}\right]=\left[\Theta_{1}\left(f_{r}\right),\Theta_{2}\left(f_{r}\right)\right]$ and $F\left\{\dot{\theta }\left(t_{r}\right)\right\}=\left[F\left\{\dot{\theta_{1}}\left(t_{r}\right)\right\},F\left\{\dot{\theta_{2}}\left(t_{r}\right)\right\}\right]=\left[{\dot{\Theta }}_{1}\left(f_{r}\right),{\dot{\Theta }}_{2}\left(f_{r}\right)\right]$, using 300 frequency values logarithmically spaced in the interval $f_{r}\in \left[10^{-2},10^{2}\right]$ Hz, where F stands for the Fourier operator. We organized the data into an N×L=(20×20)×(2×4×300)-dimensional array:

(42) W˜(f)=Re{Θ11T}Im{Θ11T}Re{Θ21T}Im{Θ21T}Re{Θ˙11T}Im{Θ˙11T}Re{Θ˙21T}Im{Θ˙21T}⋯⋯⋯⋯⋯⋯⋯⋯Re{Θ1iT}Im{Θ1iT}Re{Θ2iT}Im{Θ2iT}Re{Θ˙1iT}Im{Θ˙1iT}Re{Θ˙2iT}Im{Θ˙2iT}⋯⋯⋯⋯⋯⋯⋯⋯Re{Θ1NT}Im{Θ1NT}Re{Θ2NT}Im{Θ2NT}Re{Θ˙1NT}Im{Θ˙1NT}Re{Θ˙2NT}Im{Θ˙2NT}.

As before, after normalizing the columns of ${\tilde{W}}^{\left(f\right)}$ by the arithmetic mean and the standard deviation, we obtained the normalized array $W^{\left(f\right)}$. The rows of $W^{\left(f\right)}$, denoted by $v_{i}^{\left(f\right)}$, with i=1,⋯,N, were then used to compute the dissimilarity matrices $D_{h}=\left[d_{h}\left(v_{i}^{\left(f\right)},v_{j}^{\left(f\right)}\right)\right]$, i,j=1,⋯,400, with h=1,2, as defined by Expressions (28) and (29), and input into the MDS.

**Figure 6:**
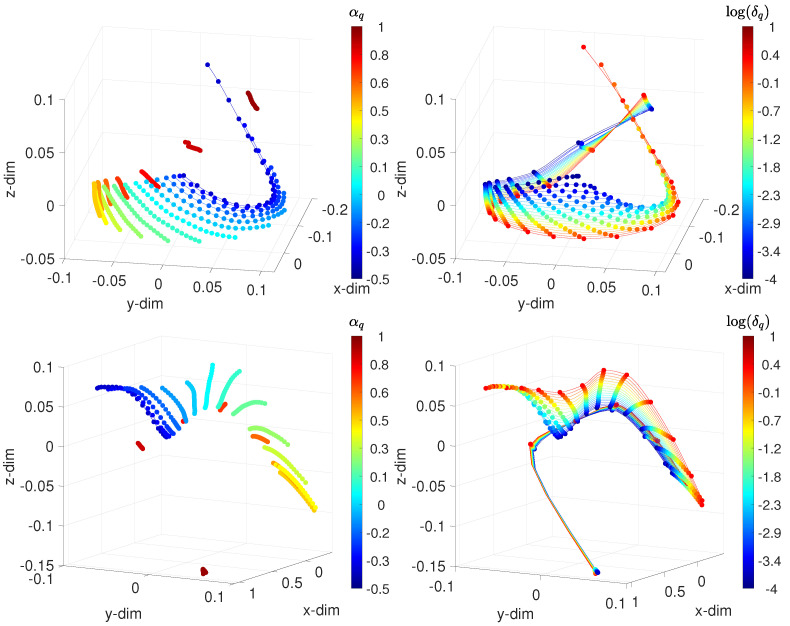
The 3-dim MDS locus for the IVSC-FSM, assessing the frequency-domain behavior, with the distances: (**top**) Arccosine $d_{1}\left(v_{i}^{\left(f\right)},v_{j}^{\left(f\right)}\right)$; and (**bottom**) Jaccard $d_{2}\left(v_{i}^{\left(f\right)},v_{j}^{\left(f\right)}\right)$. The points represent the test cases. On the left, lines connect points of constant δ, and each color corresponds to points of constant α. On the right, lines connect points of constant α, and each color corresponds to points of constant δ. The fractional order $\alpha_{q}\in \left[-0.5,1\right]$, the width of the band $\delta_{q}\in \left[10^{-4},10^{1}\right]$, and $f_{r}\in \left[10^{-2},10^{2}\right]$ Hz.

Figure 6 shows the MDS 3-dim loci using $\left\{d_{1},d_{2}\right\}$. As before, the points, which represent test cases, are linked by lines of constant δ (or α) values, while each color corresponds to one value of α (or δ). We verified the existence of patterns that were similar to the ones observed before the analysis, thus confirming the results.

### 3.2. Fractional Variable Structure Control and Integer Sliding Mode

We explored the FVSC-ISM, where the proportional-like VSS was replaced by a proportional-integral controller with a VSS control action in the integral part. Therefore, we included a fractional derivative in series with the VSS switching law. The switching border is given by (15), and the controller is:

(43) $$T_{k}^{VSS}=\left\{\begin{array}{cc} +D_{k}, & \sigma_{k}>\delta_{k}\\ \frac{\sigma_{k}}{\delta_{i}}D_{k}, & -\delta_{k}\leq \sigma_{k}\leq \delta_{k}\\ -D_{k}, & \sigma_{i}<-\delta_{k} \end{array}\right.,$$

(44) $$T_{k}=D^{\alpha }\left[T_{k}^{VSS}\left(t\right)\right].$$

As before, the test cases were based on changing the fractional order α=$\left\{\alpha_{1},\cdots ,\alpha_{q},\cdots \alpha_{Q}\right\}$, $\alpha_{q}\in \left[-0.2,0.6\right]$, and the width of the band $\delta_{k}=\delta$ (k=1,2) $\delta =\{\delta_{1},\cdots ,\delta_{q},\cdots \delta_{Q}\}$, $\delta_{q}\in \left[10^{-4},10^{1}\right]$. For both parameters, Q=20 equidistant values were considered, yielding N=20×20 test cases. The controller and the manipulator parameters were set equal to the ones adopted in Section 3.1 (i.e., $T=10^{-4}$, $\lambda_{k}=10$, with k=1,2, and $D_{1}=200,D_{2}=100$).

**Figure 7:**
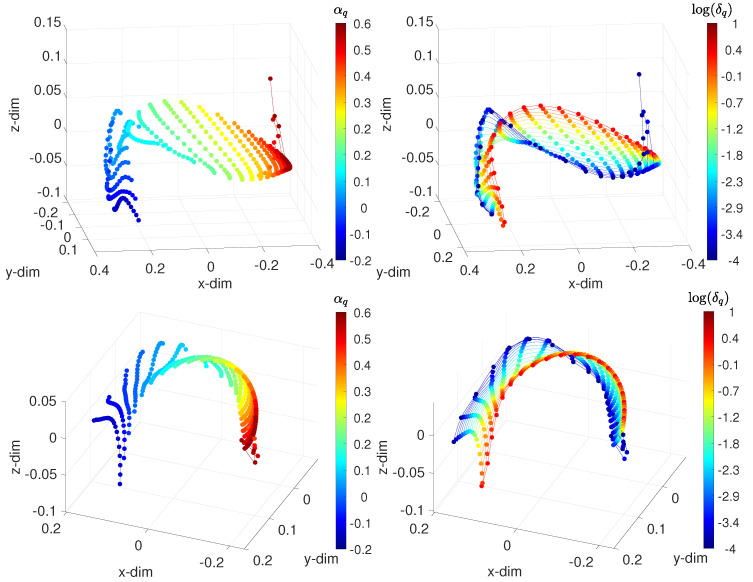
The 3-dim MDS locus for the FVSC-ISM, assessing the time domain-behavior, with the distances: (**top**) Arccosine $d_{1}\left(v_{i}^{\left(t\right)},v_{j}^{\left(t\right)}\right)$; and (**bottom**) Jaccard $d_{2}\left(v_{i}^{\left(t\right)},v_{j}^{\left(t\right)}\right)$. The points represent the test cases. On the left, lines connect points of constant δ, and each color corresponds to points of constant α. On the right, lines connect points of constant α, and each color corresponds to points of constant δ. The fractional order $\alpha_{q}\in \left[-0.2,6\right]$, and the width of the band $\delta_{q}\in \left[10^{-4},10^{1}\right]$.

**Figure 8:**
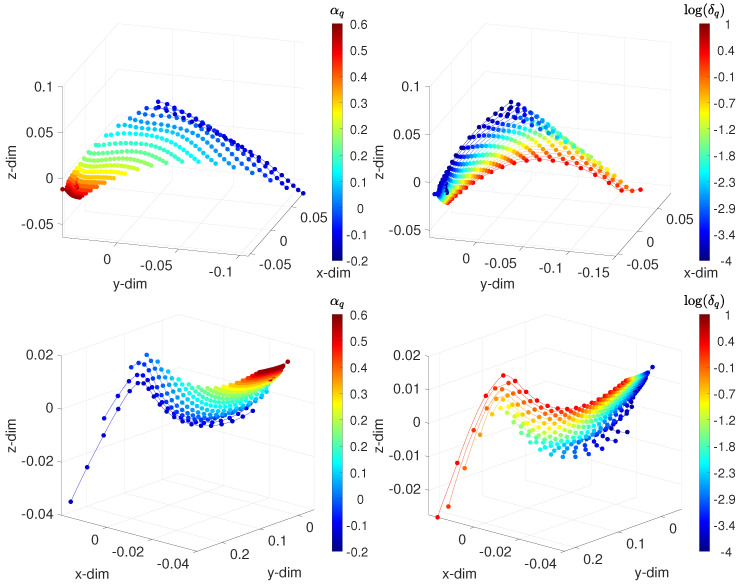
The 3-dim MDS locus for the FVSC-ISM, assessing the frequency-domain behavior, with the distances: (**top**) Arccosine $d_{1}\left(v_{i}^{\left(f\right)},v_{j}^{\left(f\right)}\right)$; and (**bottom**) Jaccard $d_{2}\left(v_{i}^{\left(f\right)},v_{j}^{\left(f\right)}\right)$. The points represent the test cases. On the left, lines connect points of constant δ, and each color corresponds to points of constant α. On the right, lines connect points of constant α, and each color corresponds to points of constant δ. The fractional order $\alpha_{q}\in \left[-0.2,0.6\right]$, the width of the band $\delta_{q}\in \left[10^{-4},10^{1}\right]$, and $f_{r}\in \left[10^{-2},10^{2}\right]$ Hz.

Figure 7 portrays the 3-dim loci generated with the distances $d_{1}\left(v_{i}^{\left(t\right)},v_{j}^{\left(t\right)}\right)$ and $d_{2}\left(v_{i}^{\left(t\right)},v_{j}^{\left(t\right)}\right)$. We verified the existence of patterns that characterized the behavior of the system as the values of the parameters varied. For constant and low α, as δ increases, the paths spread. The round trips are similar to each other for low values of α. As α increases, the inflection tends to vanish, and the locus becomes more insensitive to δ. For constant δ, patterns also emerge, and we verified that they were of the same type, independent of the values of α. Figure 8 depicts the MDS 3-dim loci using $d_{1}\left(v_{i}^{\left(f\right)},v_{j}^{\left(f\right)}\right)$ and $d_{2}\left(v_{i}^{\left(f\right)},v_{j}^{\left(f\right)}\right)$.

To sum up, the MDS technique is able to represent large sets of objects with different classes of performance indices. The interpretation of the loci is based on the emerging patterns and clusters. The goal herein was to investigate the use of MDS to assess and easily visualize the performance of fractional-order VSC applied to revolute planar robotic manipulators. Indeed, classical indices can be adopted to assess the performance. For instance, standard time-domain parameters, such as the rise, peak and settling time, overshoot, and peak value have been widely used. However, often, single (or small set) criteria are insufficient, and different indices can lead to conflicting information. Therefore, assessing control systems’ performance is a multidimensional problem that can take advantage of present-day computational information visualization techniques. The analysis adopted was based on data from the control system operation. The proposed MDS approach assumes that all relevant factors are implicitly embedded in these data. Obviously, the MDS analysis and visualization are not independent of the controlled system. However, this is not a drawback of the method. On the contrary, other systems and the influence of distinct parameters can be easily assessed by generating MDS loci that embed their corresponding information. In conclusion, the MDS constitutes a computational tool capable of generating useful representations of large sets of objects without being limited to a single class of performance indices. In this paper, the objects were closed-loop controlled systems, which differed from each other by a number of parameters. The relationships between such objects were unveiled based on the clusters and patterns that emerged on the MDS loci. More parameters can be assessed easily, generating additional objects for visualization. Therefore, further research using distinct indices and algorithms can be performed. Therefore, the results point toward further advances using other indices and fractional algorithms.

## 4. Conclusions

We addressed the use of MDS to study fractional-order VSCs. The approach compares patterns emerging in the MDS loci when varying the controller parameters. Different indices for processing the system state in the time and the frequency domains were adopted. The results illustrated the effectiveness of the MDS representation and revealed that it is simple to generate and interpret different controlled systems for a variety of test conditions. The generalization to other systems and the adoption of new assessment indices is straightforward.

## Figures and Tables

**Table 1:** Two optimal solutions determined by the PSO for N=6, yielding the fractional orders α={0.16,0.40}.

Order	Stage Elements						Parameters		
α	{$M_{i}$,$B_{i}$,$K_{i}$},k=1,⋯,6						*I*	σ	Ω
	1	2	3	4	5	6			
0.16	0.30, 0.09, 0.34	0.63, 0.33, 0.59	0.87, 0.22, 0.38	0.22, 0.98, 1.38	0.17, 0.44, 0.49	0.76, 0.77, 0.15	1.08	6.89	4.25
0.40	0.12, 0.78, 0.93	0.57, 0.22, 0.58	0.84, 0.75, 0.12	0.54, 0.08, 0.84	0.67, 0.36, 0.85	1.12, 1.15, 1.61	0.97	9.29	4.73

## References

[1] Utkin V.I. (1977). Variable Structure Systems with Sliding Modes. IEEE Trans. Autom. Control.

[2] Utkin V., Poznyak A., Orlov Y., Polyakov A. (2020). Conventional and high order sliding-mode control. J. Frankl. Inst..

[3] Shi J., Liu H., Bajçinca N. (2008). Robust control of robotic manipulators based on integral sliding mode. Int. J. Control.

[4] Gao W., Hung J.C. (1993). Variable structure control of nonlinear systems: A new approach. IEEE Trans. Ind. Electron..

[5] Veselỳ V. (1998). Decentralized variable structure control of complex systems. Int. J. Syst. Sci..

[6] Pan Y., Yang C., Pan L., Yu H. (2017). Integral sliding-mode control: Performance, modification, and improvement. IEEE Trans. Ind. Inform..

[7] Young K.K.D. (1978). Controller Design for a Manipulator Using Theory of Variable Structure System. IEEE Trans. Syst. Man Cybern..

[8] Morgan R.G., Özgüner U. (1985). A Decentralized Variable Structure Control Algorithm for Robotic Manipulators. J. Robot. Autom..

[9] Truong T.N., Vo A.T., Kang H.J. (2021). A backstepping global fast terminal sliding-mode control for trajectory tracking control of industrial robotic manipulators. IEEE Access.

[10] Su Y., Zheng C. (2020). A new nonsingular integral terminal sliding-mode control for robot manipulators. Int. J. Syst. Sci..

[11] Slotine J.J.E. (1985). The Robust Control of Robot Mattipulators. Int. J. Robot. Res..

[12] Norsahperi N., Danapalasingam K. (2020). An improved optimal integral sliding-mode control for uncertain robotic manipulators with reduced tracking error, chattering, and energy consumption. Mech. Syst. Signal Process..

[13] Soltanpour M.R., Zaare S., Haghgoo M., Moattari M. (2020). Free-chattering fuzzy sliding-mode control of robot manipulators with joints flexibility in presence of matched and mismatched uncertainties in model dynamic and actuators. J. Intell. Robot. Syst..

[14] Machado J.A.T., de Carvalho J.L.M. A New Variable Structure Controller for Robot Manipulators. Proceedings of the Third IEEE International Symposium on Intelligent Control.

[15] Huang J., Zhang M., Ri S., Xiong C., Li Z., Kang Y. (2019). High-order disturbance-observer-based sliding-mode control for mobile wheeled inverted pendulum systems. IEEE Trans. Ind. Electron..

[16] Baleanu D., Diethelm K., Scalas E., Trujillo J.J. (2016). Fractional Calculus: Models and Numerical Methods.

[17] Kenneth M., Ross B. (1993). An Introduction to the Fractional Calculus and Fractional Differential Equations.

[18] Mainardi F. (2010). Fractional Calculus and Waves in Linear Viscoelasticity: An Introduction to Mathematical Models.

[19] Luo Y., Chen Y. (2012). Fractional Order Motion Controls.

[20] Ionescu C.M. (2013). The Human Respiratory System: An Analysis of the Interplay between Anatomy, Structure, Breathing and Fractal Dynamics.

[21] Ionescu C.M., Dulf E.H., Ghita M., Muresan C.I. (2020). Robust controller design: Recent emerging concepts for control of mechatronic systems. J. Frankl. Inst..

[22] Chen L., Pan W., Wu R., Tenreiro Machado J.A., Lopes A.M. (2016). Design and implementation of grid multi-scroll fractional-order chaotic attractors. Chaos.

[23] Doha E.H., Abdelkawy M.A., Amin A.Z.M., Lopes A.M. (2019). Shifted Jacobi–Gauss-collocation with convergence analysis for fractional integro-differential equations. Commun. Nonlinear Sci. Numer. Simul..

[24] Ghita M., Neckebroek M., Juchem J., Copot D., Muresan C.I., Ionescu C.M. (2020). Bioimpedance sensor and methodology for acute pain monitoring. Sensors.

[25] Lopes A.M., Machado J.A.T. (2016). Modeling vegetable fractals by means of fractional-order equations. J. Vib. Control.

[26] Machado J.A.T., Azenha A. Fractional-Order Hybrid Control of Robot Manipulators. Proceedings of the 1998 IEEE International Conference on Systems, Man and Cybernetics.

[27] Önder Efe M., Baleanu D., Guvenc Z.B., Tenreiro Machado J.A. (2010). Fractional Order Sliding Mode Controller Design for Fractional Order Dynamic Systems. New Trends in Nanotechnology and Fractional Calculus Applications.

[28] Delavari H., Lanusse P., Sabatier J. (2013). Fractional order controller design for a flexible link manipulator robot. Asian J. Control.

[29] Delavari H., Heydarinejad H. (2018). Fractional-order backstepping sliding-mode control based on fractional-order nonlinear disturbance observer. J. Comput. Nonlinear Dyn..

[30] Fei J., Wang H., Fang Y. (2021). Novel neural network fractional-order sliding-mode control with application to active power filter. IEEE Trans. Syst. Man Cybern. Syst..

[31] Ma Z., Liu Z., Huang P. (2020). Fractional-order control for uncertain teleoperated cyber-physical system with actuator fault. IEEE/ASME Trans. Mechatron..

[32] Xie Y., Zhang X., Meng W., Zheng S., Jiang L., Meng J., Wang S. (2021). Coupled fractional-order sliding-mode control and obstacle avoidance of a four-wheeled steerable mobile robot. ISA Trans..

[33] Delavari H., Jokar R. (2021). Intelligent Fractional-Order Active Fault-Tolerant Sliding Mode Controller for a Knee Joint Orthosis. J. Intell. Robot. Syst..

[34] Wang Y., Li B., Yan F., Chen B. (2019). Practical adaptive fractional-order nonsingular terminal sliding mode control for a cable-driven manipulator. Int. J. Robust Nonlinear Control.

[35] Zhou M., Feng Y., Xue C., Han F. (2020). Deep convolutional neural network based fractional-order terminal sliding-mode control for robotic manipulators. Neurocomputing.

[36] Kumar J., Kumar V., Rana K. (2018). Design of robust fractional order fuzzy sliding mode PID controller for two link robotic manipulator system. J. Intell. Fuzzy Syst..

[37] Ahmed S., Wang H., Tian Y. (2021). Adaptive fractional high-order terminal sliding-mode control for nonlinear robotic manipulator under alternating loads. Asian J. Control.

[38] Alipour M., Malekzadeh M., Ariaei A. (2021). Practical fractional-order nonsingular terminal sliding-mode control of spacecraft. ISA Trans..

[39] Podlubny I. (1999). Fractional-order systems and *P**I*^λ^*D*^μ^-controllers. IEEE Trans. Autom. Control.

[40] Machado J.T., Galhano A. (2009). Approximating fractional derivatives in the perspective of system control. Nonlinear Dyn..

[41] De Keyser R., Muresan C.I., Ionescu C.M. (2018). An efficient algorithm for low-order direct discrete-time implementation of fractional order transfer functions. ISA Trans..

[42] Oustaloup A. (1991). La Commande CRONE: Commande Robuste D’Ordre Non Entier.

[43] Oustaloup A., Levron F., Mathieu B., Nanot F.M. (2000). Frequency-band complex noninteger differentiator: Characterization and synthesis. IEEE Trans. Circuits Syst. I Fundam. Theory Appl..

[44] Pan I., Das S. (2013). Frequency domain design of fractional order PID controller for AVR system using chaotic multi-objective optimization. Int. J. Electr. Power Energy Syst..

[45] Lopes A.M., Tenreiro Machado J., Galhano A.M. (2019). Towards fractional sensors. J. Vib. Control.

[46] Muresan C.I., Birs I.R., Dulf E.H., Copot D., Miclea L. (2021). A Review of Recent Advances in Fractional-Order Sensing and Filtering Techniques. Sensors.

[47] Ware C. (2012). Information Visualization: Perception for Design.

[48] Spence R. (2001). Information Visualization: An Introduction.

[49] Van Der Maaten L., Postma E., Van den Herik J. (2009). Dimensionality reduction: A comparative. J. Mach. Learn. Res..

[50] Tenreiro Machado J., Lopes A.M., Galhano A.M. (2015). Multidimensional scaling visualization using parametric similarity indices. Entropy.

[51] Dunteman G.H. (1989). Principal Components Analysis.

[52] Thompson B. (2005). Canonical correlation analysis. Encyclopedia of Statistics in Behavioral Science.

[53] Tharwat A., Gaber T., Ibrahim A., Hassanien A.E. (2017). Linear discriminant analysis: A detailed tutorial. AI Commun..

[54] Child D. (1990). The Essentials of Factor Analysis.

[55] France S.L., Carroll J.D. (2010). Two-way multidimensional scaling: A review. IEEE Trans. Syst. Man Cybern. Part C Appl. Rev..

[56] Lee J.A., Lendasse A., Verleysen M. (2004). Nonlinear projection with curvilinear distances: Isomap versus curvilinear distance analysis. Neurocomputing.

[57] Belkin M., Niyogi P. (2003). Laplacian eigenmaps for dimensionality reduction and data representation. Neural Comput..

[58] Coifman R.R., Lafon S. (2006). Diffusion maps. Appl. Comput. Harmon. Anal..

[59] Van der Maaten L., Hinton G. (2008). Visualizing data using t-SNE. J. Mach. Learn. Res..

[60] McInnes L., Healy J., Melville J. (2018). UMAP: Uniform manifold approximation and projection for dimension reduction. arXiv.

[61] De Oliveira E.C., Machado J. (2014). A review of definitions for fractional derivatives and integrals. Math. Probl. Eng..

[62] Dorčák V. (2002). Numerical models for the simulation of the fractional-order control systems. arXiv.

[63] Podlubny I. (1999). Functional Differential Equations.

[64] Al-Alaoui M.A. (2008). Al-Alaoui operator and the new transformation polynomials for discretization of analogue systems. Electr. Eng..

[65] Lopes A.M., Machado J.T. (2021). Multidimensional scaling analysis of generalized mean discrete-time fractional order controllers. Commun. Nonlinear Sci. Numer. Simul..

[66] Machado J.A.T. (1996). Variable Structure Control of Manipulators with Joints having Flexibility and Backlash. Syst. Anal. Model. Simul..

[67] Azenha A., Machado J.A.T. Dynamic Analysis in Variable Structure Position/Force Hybrid Control of Manipulators. Proceedings of the 1997 IEEE International Conference on Systems, Man and Cybernetics.

[68] Machado J.T. (2012). The effect of fractional order in variable structure control. Comput. Math. Appl..

[69] Machado J.A.T., de Carvalho J.M., Galhano A. (1993). Analysis of Robot Dynamics and Compensation Using Classical and Computed Torque Techniques. IEEE Trans. Educ..

[70] Merrikh-Bayat F., Afshar M., Karimi-Ghartemani M. (2009). Extension of the root-locus method to a certain class of fractional-order systems. ISA Trans..

[71] Lopes A.M., Tenreiro Machado J. (2013). Root locus practical sketching rules for fractional-order systems. Abstr. Appl. Anal..

[72] Biswas K., Bohannan G., Caponetto R., Lopes A., Machado T. (2017). Fractional Order Devices.

[73] Petráš I. (2009). Fractional-order feedback control of a DC motor. J. Electr. Eng..

[74] Ionescu C.M., Muntean I., Tenreiro-Machado J., De Keyser R., Abrudean M. (2010). A theoretical study on modeling the respiratory tract with ladder networks by means of intrinsic fractal geometry. IEEE Trans. Biomed. Eng..

[75] Daou R.A.Z., Francis C., Moreau X. (2009). Synthesis and implementation of noninteger integrators using RLC devices. Int. J. Electron..

[76] Deza M.M., Deza E. (2009). Encyclopedia of Distances.

